# Review of Industry Payments to General Orthopaedic Surgeons Reported by the Open Payments Database: 2014 to 2019

**DOI:** 10.5435/JAAOSGlobal-D-21-00060

**Published:** 2021-05-07

**Authors:** Johann Braithwaite, Nicholas Frane, Matthew J. Partan, Peter B. White, Cesar Iturriaga, Joshua Gruber, Adam Bitterman

**Affiliations:** From the Zucker School of Medicine at Hofstra/Northwell Orthopaedic Surgery Residency Program, Plainview, NY (Dr. Braithwaite, Dr. Frane, Dr. Partan, Dr. White, and Dr. Bitterman), the Department of Orthopaedic Surgery, Northwell Health Huntington Hospital, Huntington, NY (Dr. Braithwaite, Dr. Frane, Dr. Partan, Dr. White, Dr. Iturriaga, and Dr. Bitterman), Nova Southeastern University (Mr. Gruber).

## Abstract

**Introduction:**

The Physician Payments Sunshine Act was placed into law in 2010 in an effort to create transparency between physicians and industry. Along with many other specialties, orthopaedic surgeons have long worked intimately with medical industry companies. This study aimed to evaluate trends in industry payments to general orthopaedic surgeons from 2014 to 2019.

**Methods:**

A retrospective review of the Center of Medicare and Medicaid Services' Open Payments Database was done to identify all industry payments to all general orthopaedic surgeons (ie, not subspecialty affiliated) from 2014 to 2019. The researchers analyzed total payments and subtype payments for yearly trends, and a regional analysis was done. The primary outcome was the overall trend in total median payments, which was assessed through the Jonckheere-Terpstra test. Descriptive statistics include medians with interquartile ranges. *P* < 0.05 was considered statistically significant.

**Results:**

Between 2014 and 2019, a total of 1,330,543 payments totaling $1.79 billion dollars was paid to 108,041 general orthopaedic surgeons. During this time, the number of surgeons receiving payments increased with a significant uptrend in median payments per surgeon (*P* < 0.001; Table 1). The top 25% percentile of general orthopaedic surgeons received >95% of payments, whereas the bottom 25% received <0.1%. The general payment types all saw significant increases (*P* < 0.001) between 2014 and 2019, with the exception of “Ownership or Investment Interests” (*P* = 0.657) and “Royalty or License” (*P* = 0.517). Significant regional uptrends in median industry payments were also seen in the Midwest, Northeast, South, and West (*P* < 0.001). Four of the top five orthopaedic industry companies made payment increases between 2014 and 2019.

**Conclusion:**

Industry payments to general orthopaedic surgeons between 2014 and 2019 have increased with a considerable disparity in payments among the top-paid orthopaedic surgeons.

In recent times, the close relationship between physicians and the medical industry has raised the concern that the medical industry can influence physician decisions.^1^ The Physician Payments Sunshine Act (PPSA) was first established in 2010 as part of the Patient Protection Affordable Care Act, with the intent to create transparency among such relationships. The law required that all medical device, drug, and biologic companies begin reporting all transfers of value to physicians or teaching hospitals to the Centers for Medicaid and Medicare (CMS). “Transfers of value” by the medical industry companies have been termed “industry payments.” The goals of the PPSA included the following: (1) shed light on “privatized” financial relationships between physicians and industry, (2) prevent industry influence on physician decisions concerning patient care and conflicts of interest, and (3) make this information readily available for patients.^1^ Despite its noble intentions, the PPSA has also been viewed as a potential catalyst to discourage relationships between physicians and industry. In August 2013, CMS began collecting data and had since gathered and published 7 years of data on the Open Payments Database (OPD), equivalent to 76 million reported payments and $53 billion in payments to physicians and teaching hospitals. In 2016, Agrawal et al^2^ accurately reflected the strength of the physician-industry relationships by demonstrating payments totaling $3.4 billion to 470,000 physicians and 1,019 teaching hospitals.

Among all specialties, orthopaedic surgery has maintained one of the most substantial industry relationships because of its extensive use of technology, devices, or implants.^3,4^ Orthopaedic surgeons regularly work intimately with industry representatives or companies to research and develop new technology. In the first data set reported by the CMS in 2014, orthopaedic surgeons only accounted for 3.4% of the 360,000 physicians studied but received almost 20% of the overall payments and nearly 25% of the general payments made to physicians.^1^ In a 2016 analysis, general orthopaedic surgeons have been shown to account for nearly 80% of all industry payments to orthopaedic surgeons in the OPD.^5^ Despite the overpowering contribution of general orthopaedic surgeons, the previous literature examining the PPSA in orthopaedics has narrowly focused not only on isolated years of the OPD but purely on specific orthopaedic subspecialties.^6-8^

Given the lack of literature exploring the effects of the PSSA on general orthopaedic surgeons, a more global understanding of their industry relationships is crucial. To our knowledge, no studies have evaluated the effects of general payments made to general orthopaedic surgeons.

The aim of this study was to (1) analyze trends in industry payments to general orthopaedic surgeons from 2014 to 2019, (2) analyze trends of various subtype payments, (3) compare median payments per region (ie, Northeast, Southeast, Southwest, and Northwest), and (4) compare the payments among the top five industry payment companies. We hypothesized that there would be an increase in the total number of general payments made by industry because of an overall increase in the number of general orthopaedic surgeons reported on the OPD.^6,9^ We also hypothesized no change in the median payments made per surgeon, as demonstrated in previous studies.^6-8^

## Methods

A retrospective review of the CMS Open Payments Database was done to identify all payments to general orthopaedic–trained orthopaedic surgeons between 2014 and 2019. The CMS Open Payments database includes all physicians from the United States who received at least one industry payment or transfer of value worth at least $10 or received payments totaling more than $100 in a calendar year. The CMS generally tasked the company representatives with reporting the industry payments and assigning the specialty/subspecialty.

To conduct this study, the data set was stratified to identify all payments to orthopaedic surgeons. The data set was further stratified based on orthopaedic subspecialty, and all “Allopathic & Osteopathic Physicians|Orthopaedic Surgery” payments were selected and combined by year to reflect total payments paid to surgeons (Table 1). The OPD has released seven data sets, including the last 5 months of 2013 and the full years of 2014 to 2019. For this study, we excluded the data from 2013 to avoid seasonal and confounding variables. Across the study period (2014 to 2019), a total of 1,308,643 payments (68.0% of all orthopaedic payments) were made to 29,179 general orthopaedic surgeons, which comprised 68.5% of all orthopaedic surgeons listed in the OPD. We excluded surgeons licensed under a subspecialty from this study. No other exclusion criteria were used. We stratified each surgeon for 1-year summaries of their payments. Each year was counted separately across the 6-year study period.

**Table 1:** Total Trends for Increasing Yearly Payments to General Orthopaedic Surgeons

Year	No. Payments	Payment Sum ($)	Surgeons (N)	Median Yearly Payment ($)	Percentile (25th)	Percentile (75th)	*P*^a^
2014	215,439	260,449,945.85	17,861	292.54	80.48	1,475.8	—
2015	209,809	250,674,718.30	17,372	318.90	86.65	1,600.16	0.017^a,b^
2016	218,134	293,897,348.24	17,285	366.15	94.2	1,926.65	0.000^a,b^
2017	216,647	292,884,399.36	18,800	339.27	89.87	1,876.51	0.000^a,b^
2018	223,720	304,472,984.29	18,350	427.23	104.7	2,204.31	0.000^a,b^
2019	246,794	394,133,594.07	18,373	508.66	115.83	2,467.84	0.000^a,b^

A Jonckheere-Terpstra test for ordered alternatives demonstrated increase median yearly payment with increasing years (*P* < 0.001).

a Mann-Whitney *U* test compared with 2014.

b Statistical significance reached.

$ = United States Dollars.

The study's primary outcome was to evaluate overall yearly trends in general payment per surgeon from 2014 to 2019. Secondary outcomes included analyzing various subtype payments, comparing median payments per region (ie, Northeast, Southeast, Southwest, and Northwest) and comparing the top five industry payment companies' payments. Subtype payments included in the database included charitable contributions, faculty or speaking fees, consulting fees, ownership or investment payments, educational payments, entertainment/food and beverage payments, Gifts, Grants, Honoraria, royalties, and travel and lodging. Regional areas were determined based on the US census regions. The top five industry payment companies were selected based on an analysis of all orthopaedic companies' annual contributions reporting to the OPD.

Data analysis was done with SPSS version 26 (IBM). A *P*-value of less than 0.05 was considered statistically significant. The Shapiro-Wilks test of normalcy was done on all data, which was found to be nonparametric (*P* < 0.001). The primary outcome was the overall trend in total median payments to general orthopaedic surgeons from 2014 to 2019, assessed through the Jonckheere-Terpstra test. In addition, we considered the first year of payments (2014) as an index year and compared all subsequent years pairwise with the index year through a Mann-Whitney *U* test. Subsequent trend analyses on the number of surgeons compensated per year, payment subtype, and regional distributions were done using the Jonckheere-Terpstra test. Descriptive statistics presented include medians with interquartile ranges.

## Results

### Annual Trends of General Payments

Between 2014 and 2019, the number of general orthopaedic surgeons receiving payments increased from 17,861 to 18,373. The median payment value increased from $292.54 to $508.66 (USD) between 2014 and 2019 (*P* < 0.001). The top 25% of surgeons received payments of at least $1,475.80 in 2014, and this is increased to $2,467.84 in 2019. The bottom 25% of surgeons received payments of at most $80.48 in 2014, which is increased to $115.83 in 2019 (Table 1).

**Figure 1:**
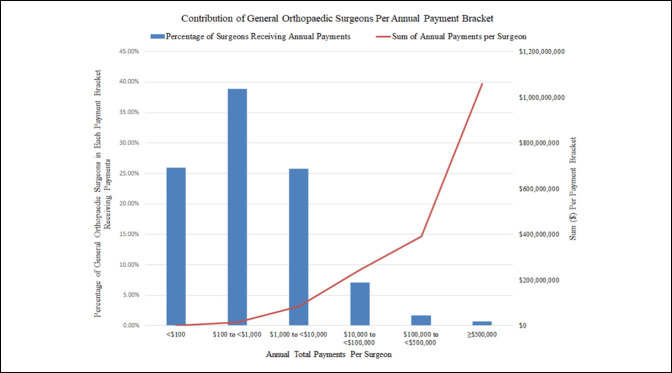
Percentage of surgeons receiving annual payments and annual payment sum per surgeon within payment brackets.

From 2014 to 2019, a total of $1,796,535,874.93 was paid to general orthopaedic surgeons. A general increase was observed in the total amount paid over the years of the study, increasing from $260,472,564.51 to $394,133,594.07. A net increase was also observed in the percent of the total payments made to the five highest-paid surgeons from 10.18% of total payments (2014) to 22.96% (2019) (Figure 1).

**Figure 2:**
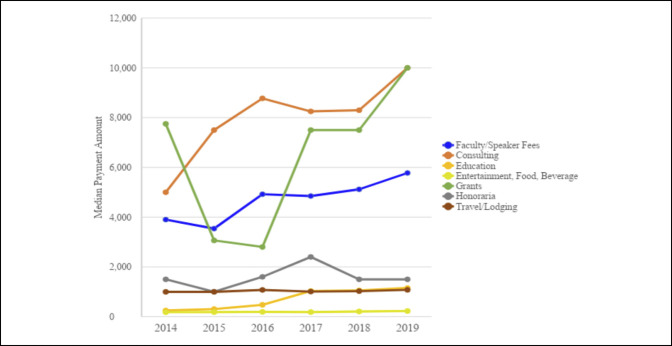
Bar chart showing percentage of surgeons receiving annual payments versus sum of payments per surgeon per payment bracket.

Between 2014 and 2019, 25.9% (27,947) of general orthopaedic surgeons received a total yearly average payment <$100 for a sum of $1,180,113.74 (0.1%). The general orthopaedic surgeons receiving average payments between $100 and $1,000 (38.9% of the population) received $15,051,707.46 (0.8%) of payments. The surgeons receiving average payments between $1,000 and $10,000 (25.8% of the population) received 4.7% of the total payments. More than 94% of the total payments between 2014 and 2019 were paid to 10,259 (9.5%) general orthopaedic surgeons receiving average annual payments > $10,000 (Table 2, Figure 2).

**Table 2:** Disparity of the General Payments Made to General Orthopaedic Surgeons

Yearly Total Payment	Surgeons (n)	Percent of Total Surgeons	Sum Contributions	Percent of Sum Contributions
<$100	27,947	25.90	1,180,113.74	0.10
$100-1,000	42,009	38.90	15,051,707.46	0.80
$1000-10,000	27,626	25.80	84,253,475.20	4.70
$10,000-100,000	7,697	7.10	245,249,560.93	13.70
$100,000-<500,000	1,826	1.70	390,774,483.81	21.80
>$500,000	736	0.70	1,060,026,533.79	59.00

**Figure 3:**
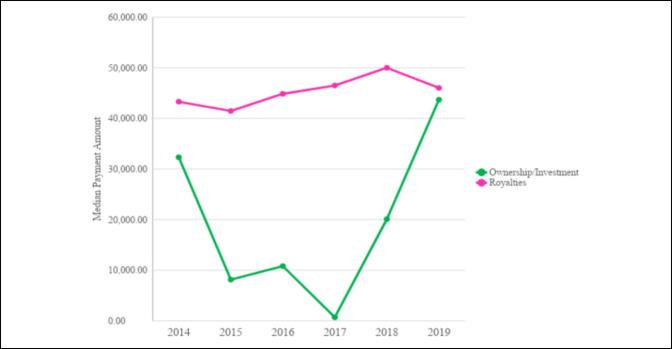
Line chart showing lower median payment subtypes.

**Figure 4:**
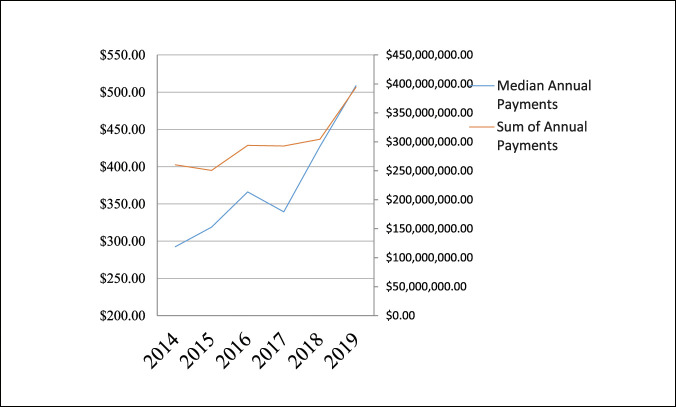
Line chart showing higher median payment subtypes with no notable annual increase.

Analysis of general payment subtypes revealed a trend of increasing median payment values from 2014 to 2019 in faculty/speaker fees (*P* < 0.001), consulting fees (*P* < 0.001), education (*P* < 0.001), entertainment/food/beverages (*P* < 0.001), gifts (*P* < 0.001), grants (*P* = 0.002), honoraria (*P* < 0.001), and traveling/lodging (*P* < 0.001). There was no evidence of a trend of increasing median payment value over the years in ownership/investment interest (*P* = 0.657) or in royalty/license (*P* = 0.517). From 2014 to 2019, no significant decrease was observed in median payment value in charitable donations from $5084.75 to $250.00 (*P* = 0.672) (Table 3, Figure 3, Figure 4).

**Table 3:** Annual Median Payments by Nature of Payment

Payment Subtype	2014		2015		2016		2017		2018		2019		*P*
	Median	IQR	Median	IQR	Median	IQR	Median	IQR	Median	IQR	Median	IQR	
Faculty or speaker fees	3,900	12,500	3,540	11,425	4,920.33	12,125	4,848	12,900	5,118.75	13,513.88	5,775	14,125	<0.001
Consulting fee	5,000	17,500	7,500	23,369.24	8,775	24,425	8,250	21,637.50	8,296.92	23,800	10,000	25,106.67	<0.001
Ownership or investment interest	32,307.37	109,986	8,135	109,784.82	10,800	91,560	687.6	24,387	20,100	61,206	43,690.46	165,864.03	0.657
Education	244.26	545.72	303.48	871.96	474.99	1,259.75	1,029	1,846.01	1,052	1,850.01	1,148.50	1,796.98	<0.001
Entertainment, food, and beverage	177.83	374.24	181.68	389.76	192.01	410.75	182.59	383.69	207.33	435.79	227.29	486.81	<0.001
Grant	7,749.84	11,035.12	3,062.50	8,750	2,800	9,325	7,500	9,187.46	7,500	8,350	10,000	18,283	0.002
Honoraria	1,500	2,710	1,000	2,950	1,600	2,200	2,400	3,387.50	1,500	3,675	1,500	4,080	<0.001
Royalty or license	43,293.93	183,432.34	41,460.33	180,535.08	44,854.45	194,499.06	46,504.44	233,570.88	50,000	190,810.98	46,022.47	218,928.08	0.517
Travel and lodging	996.1	1,543.24	997.2	1,424.34	1,073.57	1,613.53	1,007.84	1,401.27	1,024.28	1,468.56	1,075.19	1,617.81	<0.001

### Comparison of Median Payments by Region Per Year

Comparison of regional trends demonstrates a trend of increasing median payments from 2014 to 2019 in the Midwest from $328.41 to $576.52 (*P* < 0.001), Northeast from $271.50 to $348.13 (*P* < 0.001), South from $249.40 to $489.30 (*P* < 0.001), and West from $277.91 to $425.78 (*P* < 0.001). There was no proof of a trend of significantly increasing median payments in other regions of the United States (*P* = 0.896) (Table 4).

**Table 4:** Regional Trends in General Payments in the United States

	Year																		*P*
	2014			2015			2016			2017			2018			2019			
	Median	75th P*	25th P*	Median	75th P*	25th P*	Median	75th P*	25th P*	Median	75th P*	25th P*	Median	75th P*	25th P*	Median	75th P*	25th P*	
Other	228.28	1508.93	75.87	349.41	1527.65	75.42	401.5	1880.7	96.51	175.32	1522.98	75.32	228.53	1763.07	78.98	276.28	1733.46	71.14	0.896
Midwest	328.41	1445.55	89.27	356.47	1604.97	95.88	398.86	1892.89	100.32	392.79	2023.27	102.3	511.27	2257	112.52	576.52	2324.08	115.99	<0.001
Northeast	271.5	1362.77	77.35	304.18	1409.37	85.17	339.3	1688.77	96.94	310.39	1679.37	89.8	341.96	1886.11	101.66	348.13	2019.31	100.79	<0.001
South	249.4	1304.03	70.67	284.57	1485.25	76.32	318.61	1817.14	82.74	298.85	1723.13	79.63	379.99	2054.27	92.97	489.32	2437.42	112.78	<0.001
West	277.91	1572.45	71.49	285.26	1688.1	75.41	322.41	1895.31	83.93	286.52	1679.73	76.93	380.8	2054.73	90.46	425.78	2199.56	106.27	<0.001

P* = Percentile.

Units: US Dollars.

### Comparison of Median Payments by Top Industry Companies

**Figure 5:**
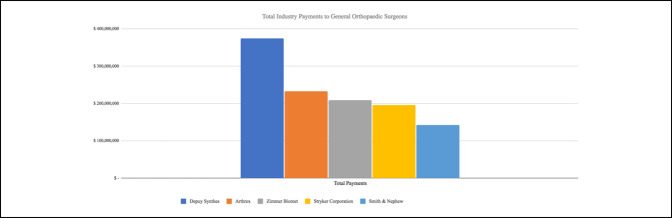
Bar diagram showing total payments 2014 to 2019 by the top five companies.

Four of the top five orthopaedic industry companies made payment increases between 2014 and 2019. Payments made by Arthrex increased by 98.1% between 2014 and 2019, Stryker Corporation increased by 34.6% between 2014 and 2019, Zimmer Biomet Holdings increased by 30.8% between 2015 and 2019, and DePuy Synthes Products LLC increased by 30.8% between 2014 and 2018. The total sum of the top five contributing companies is shown in Figure 5.

## Discussion

The PPSA was implemented with noble intentions to shed light on previously privatized industry relationships that may influence patient care and make this information readily available for patients. There have been conflicting waves of support and criticism from those intimately involved. However, industry relationships have been the primary source of funding for research and development since the start of this century.^10^ Orthopaedic technology is continuously evolving, and orthopaedic surgeons may be intimately involved in developing or training new technology with the goal of improving patient care.

Regrettably, several initial studies of nonorthopaedic specialties demonstrated some negative effects of the PPSA. They reflexively coaxed authors to explore the effects of the PPSA on industry payments but have continued to demonstrate conflicting results. The adverse effects of the legislation were first underscored by a notable decrease in the number of plastic surgeons involved in industry financial relationships with an associated reduction in the total sum of payments from 2013 to 2014.^11^ From 2014 to 2017, Rhee et al^12^ found that general payments received by nonorthopaedic surgeons declined in value. Similarly, an early decline in 2014 physician-industry transactions was also seen among medical oncologists.^13^ Industry payments to urologists from 2014 to 2018 have not had any notable changes, and a decline was not noted.^14^ On the contrary, prior authors have also produced literature that has supported continued positive relationships despite the enactment of the PPSA. Through analysis of OPD data and payment trends in orthopaedic subspecialties, specifically foot and ankle, spine, and pediatric orthopaedics, each demonstrated a lack of adverse effects on the surgeon-industry relationship.^6-8^ Notably, they showed increases in the total number of compensated surgeons, no decrease in median payment per surgeon, and increases in median payments to the top 5% of surgeons.^6,7,9^ and increases in median payments to the top 5% of surgeons.^7,9^ On reviewing the conflicting literature, we hypothesized that there would be an increase in the total number of general payments made by industry because of an overall increase in the number of general orthopaedic surgeons reported on the OPD.^6,9^ We also hypothesized no change in the median payments made per surgeon, as demonstrated in previous studies.^6,7^

Previous studies have generally looked at the general distribution of payments statically over individual years, and there has been a paucity of literature analyzing the evolution of such payments over time. In addition, the previous literature has focused on orthopaedic subspecialists with a failure to explore the effects of the OPD on general orthopaedic surgeons, who undoubtedly play a substantial role in industry payments. This is the first study to use all available data from 6 years of OPD reporting (2014 to 2019) to analyze the effects of the PPSA on general orthopaedic surgeon-industry relationships. Our results demonstrate that industry payments to general orthopaedic surgeons have continued to increase. This finding was evident in all payment subtypes and in most regions of the United States. In addition, there is an evident disparity of payments between the top-paid orthopaedic surgeons and the rest of the population.

Our 6-year trend analysis shows that the median payment per general orthopaedic surgeon consistently increased between 2014 and 2019. Further supporting this notion, there were also an increase in the total number of general orthopaedic surgeons receiving payments (17,861 in 2014 versus 18,373 in 2019) and an increase in the number of annual payments (215,439 in 2014 versus 246,794 in 2019). However, more consistent recording and reporting by industry or general orthopaedic surgeons can potentially explain these findings. We also found a disproportionate increase in payments between the bottom 25th percentile (143.9%) and the top 75th percentile (167.2%) of orthopaedic surgeons. Most notably, across the 6-year study period, a notable overall increase was observed in median industry payments per surgeon (173.9%). This study demonstrates continued growth in the relationship between general orthopaedic surgeons and industry.

The findings in our research agree with the previous literature demonstrating an apparent disparity of the industry payments made to general orthopaedic surgeons^12,15^ and nonorthopaedic surgical specialties.^12^ Pathak et al^7^ demonstrated that the top 10% of orthopaedic spine surgeons received 89% of the total general payments in 2014. The top 5% received 79%, and the top 1% received 55%. A similar study demonstrated that the top 5% of orthopaedic sports surgeons received 90.5% of payments in 2015.^16^ This study demonstrated that the top 0.6% of general orthopaedic surgeons received 64.9% of the total general payments made and the top 11% received 95% of the total general payments. This was true for all years studied. Of note, the most common payments were between $100 and $1000, and payments <$100 were the second most common for all years. On additional analysis, we found that royalty/license payments make up the most notable portion of total payments made to general orthopaedic surgeons, which agrees with previous studies of various subspecialties of orthopaedic surgery.^1,5,6,7,8,15^ Analysis of our data revealed that similar to the total median industry payments, most of royalty/licensing payments were being made to a relatively small fraction of general orthopaedic surgeons. Iyer et al^15^ demonstrated that a small fraction of orthopaedic surgeons (1.6%) received large payments (75.5% of value) for royalties and licensing fees. Our results show that the most recent year evaluated, 2019, had the highest median royalty/license payments ($46,022.47) and accounted for 38.36% of that year's sum contributions. Furthermore, the surgeons in the 75th percentile or higher accounted disproportionately for >96% of royalty/license payments in 2019. This illustrates a broad thriving relationship between general orthopaedic surgeons and industry, albeit with a smaller group of elite surgeons prospering further from the continued partnerships.

The literature on payments made by various companies between 2014 and 2019 is limited; thus, this study's authors conducted an analysis to better understand the increases in industry payments to general orthopaedic surgeons across the years. We found that most companies made increasing payments throughout the years studies. The top five companies accounted for 77.24% of total payments in 2015. Lopez et al^5^ demonstrated the same top five companies in 2013. Of the data available, these companies with the highest payments demonstrated increases in payments with their largest payments in 2019. Only one of the top five companies saw an overall decrease in payments between 2014 and 2019. It was suspected that the increased transparency of the PPSA would discourage companies from making payments to physicians. However, among the 22 companies analyzed, only three seem to have decreased payments to general orthopaedic surgeons between 2014 and 2019. Furthermore, analysis of the regional impact of the PPSA demonstrated increases in median payments in the Midwest, Northeast, South, and West, with the most notable growth seen in the South. A previous study demonstrated that among orthopaedic surgery residents receiving payments, 27% were from states where legislation was more restrictive, whereas 73% were from states that lacked additional restrictions.^17^ Although this may help to explain some of the regional differences seen over the years, additional research is necessary to determine what other factors, if any, have contributed toward these differences as well.

Notable concern raised by critics of the PPSA was that it could have an adverse impact on patient care, clinical practice, and research.^5^ A study found that physicians receiving high payment from the industry were believed to be less honest and committed to the patient's best interest.^18^ Although the media and some political groups have suggested that patients may lose trust in their physicians on learning about their industry relationships,^19^ the peer-reviewed literature has demonstrated that the general public^20^ and patients^21^ have a positive perception of surgeon-industry relationships. Furthermore, patients report considering surgeon-industry relationships to be an essential factor in choosing their surgeon despite their level of unawareness.^15^ Iyer et al^22^ demonstrated that industry relations did not negatively affect a patient's perception of their surgeon, and most payment types positively influenced >50% of patients' perceptions. Although there is a small possibility that the PPSA will negatively affect perception, there is evidence that the positive impact of the PPSA outweighs the negative.

A second primary concern is the value of reporting and the cost of maintaining the OPD. Our data demonstrated that 59.5% of payments were <$1,000, and it only accounted for 0.6% of the total payments in 2019. Furthermore, 25.9% of payments were <$100 and accounted for 0.1% of the total payments. Therefore, of the total 1,308,643 total payments made to general orthopaedic surgeons, 778,642 payments were <$1,000, and 338,938 payments were <$100. Although the cost of maintaining the OPD is unknown, it can undoubtedly be argued that resources will be saved with a revision of the rules of reporting to the OPD. Additional analysis should be done to assess the merit in reporting smaller payments to the OPD.

This study has some critical limitations aside from those stemming from its retrospective nature. Although this study succeeded in demonstrating trends in industry payments to general orthopaedic surgeons, it does not demonstrate the effect of industry payments on patient care. Our results and analysis are limited by the accuracy and inclusivity of the OPD data. The OPD's data have been questioned in the previous literature because of its dependency on honest, consistent disclosure.^23,24,25,26^ Studies have demonstrated inconsistent reporting in orthopaedic joint surgeons and general orthopaedic surgeons,^27^ and orthopaedic sports medicine surgeons.^28^ On a more honorable note, a review of the consistency and accuracy of reporting to the OPD in 2015 found less than 1% were missing mandatory data elements.^29^ A study of shoulder arthroplasty publications demonstrated that 93% of royalty payments were reported.^30^ Second, the OPD classifies payment specialty/subspecialty based on medical licensure/national provider identifier as registered with CMS. This cohort of patients includes nearly 70% of orthopaedic surgeons within the database, which is similar to what others have found,^5^ and may include subspecialty-trained physicians who are not registered with CMS as such. Regardless, this cohort composes the largest group of orthopaedic payments studied to date. Finally, our analysis did not focus on discrepancies in payment between genders among orthopaedic surgeons. A study by Ray et al^31^ demonstrated a large disparity in payments being made between genders, with 99.6% of payments being made to male physicians. The disparity in genders has also been shown in leadership roles in orthopaedic surgery.^32-34^ Orthopaedic surgery has been historically male predominant; however, the number of women entering the field is on the rise, and with the increasing presence of women in the field, the need to address these inequalities is becoming more critical. Although the OPD presents some limitations, it is currently the largest data set of physician-industry relationships available, and the authors believe that it is a large enough sample of data to accurately fulfill its intended purpose. However, additional revisions need to be made to the rules of reporting and the OPD itself to improve its efficacy and accuracy.

## Conclusion

This study demonstrated that despite increased transparency of PPSA, industry payments to general orthopaedic surgeons have continued to increase. This finding was found in all payment subtypes and in all regions in the United States. There is also an evident disparity in payments between the top-paid orthopaedic surgeons and the rest of the population.
